# A 3-dimensional *in vitro *model of epithelioid granulomas induced by high aspect ratio nanomaterials

**DOI:** 10.1186/1743-8977-8-17

**Published:** 2011-05-18

**Authors:** Vanesa C Sanchez, Paula Weston, Aihui Yan, Robert H Hurt, Agnes B Kane

**Affiliations:** 1 Department of Pathology and Laboratory Medicine, Brown University, Providence, RI, USA; 2 Molecular Pathology Core Facility, Brown University, Providence, RI, USA; 3 School of Engineering, Brown University, Providence, RI, USA

## Abstract

**Background:**

The most common causes of granulomatous inflammation are persistent pathogens and poorly-degradable irritating materials. A characteristic pathological reaction to intratracheal instillation, pharyngeal aspiration, or inhalation of carbon nanotubes is formation of epithelioid granulomas accompanied by interstitial fibrosis in the lungs. In the mesothelium, a similar response is induced by high aspect ratio nanomaterials, including asbestos fibers, following intraperitoneal injection. This asbestos-like behaviour of some engineered nanomaterials is a concern for their potential adverse health effects in the lungs and mesothelium. We hypothesize that high aspect ratio nanomaterials will induce epithelioid granulomas in nonadherent macrophages in 3D cultures.

**Results:**

Carbon black particles (Printex 90) and crocidolite asbestos fibers were used as well-characterized reference materials and compared with three commercial samples of multiwalled carbon nanotubes (MWCNTs). Doses were identified in 2D and 3D cultures in order to minimize acute toxicity and to reflect realistic occupational exposures in humans and in previous inhalation studies in rodents. Under serum-free conditions, exposure of nonadherent primary murine bone marrow-derived macrophages to 0.5 μg/ml (0.38 μg/cm^2^) of crocidolite asbestos fibers or MWCNTs, but not carbon black, induced macrophage differentiation into epithelioid cells and formation of stable aggregates with the characteristic morphology of granulomas. Formation of multinucleated giant cells was also induced by asbestos fibers or MWCNTs in this 3D *in vitro *model. After 7-14 days, macrophages exposed to high aspect ratio nanomaterials co-expressed proinflammatory (M1) as well as profibrotic (M2) phenotypic markers.

**Conclusions:**

Induction of epithelioid granulomas appears to correlate with high aspect ratio and complex 3D structure of carbon nanotubes, not with their iron content or surface area. This model offers a time- and cost-effective platform to evaluate the potential of engineered high aspect ratio nanomaterials, including carbon nanotubes, nanofibers, nanorods and metallic nanowires, to induce granulomas following inhalation.

## Background

Carbon nanotubes (CNTs) are a commercial success of nanotechnology with an expanding global market and potential applications for biomedical devices, imaging, drug delivery, tumor targeting, electronics, composite materials and sensing applications [1]. The continued economic success of this industry is threatened by uncertainty and controversy surrounding potential adverse environmental and human health effects of engineered nanomaterials [2].

CNTs have been shown to induce significant toxicity using *in vitro *cellular assays and several *in vivo *rodent bioassays [3]. In rodent bioassays using intratracheal instillation [4] or pharyngeal aspiration [5], multiwalled carbon nanotubes (MWCNTs) produced significant lung injury, epithelioid granulomas, and persistent interstitial inflammation and fibrosis. Granulomas are aggregates of epithelioid macrophages elicited by biopersistent irritants or microorganisms that are not killed during the acute inflammatory response [6]. Two major types of granulomas are non-immune or foreign body granulomas (FBG) and antigenic or immunologic granulomas [6,7]. Foreign body granulomas are characterized by aggregated epithelioid macrophages and multinucleated giant cells [8]. If the stimulus is biopersistent, granulomas may persist for months or years and lead to tissue destruction and fibrosis [7].

A major controversy in nanotoxicology is whether the observed induction of lung inflammation and fibrosis following the bolus delivery of MWCNTs by intratracheal instillation or pharyngeal aspiration is an artifact associated with agglomeration of the carbon nanotubes prior to administration [5,9]. However, a recent inhalation study [10] and a pharyngeal aspiration study using commercial samples of well-dispersed MWCNTs also induced significant lung inflammation characterized by multifocal granulomas [11]. The specific physicochemical properties of carbon nanotubes responsible for lung toxicity are unknown. It is uncertain whether these pathologic responses are due to bioavailable metal catalyst residues, graphitic or amorphous carbon residues, length, geometry, high surface area, three-dimensional structure, agglomeration state, or some unique property of the carbon nanotube graphenic surface [12,13].

There are numerous animal models of immune-mediated granulomas. *In vitro *models of immune-mediated granulomas have been induced by exposure of macrophages to pathogens or pathogen-coated beads. Macrophages exposed to heat-killed *Candida albicans *[14], *Mycobacterium sp*. [15], parasites such as *Nippostrongylus brasiliensis *[16], or antigens from *Schistosoma sp*. eggs bounded to 45 μm latex beads [17], produce granulomas *in vitro*.

Although non-degradable particles such as latex or dextran beads can induce foreign body or non-immune granulomas in monolayer cultures of macrophages [17], there is no *in vitro *model that recreates granuloma formation induced by biopersistent, high aspect ratio nanomaterials. To comply with a mandate issued by the U.S. National Academy of Sciences to replace animal testing with high-throughput *in vitro *cellular assays based on mechanistic pathways [18], we have developed a novel *in vitro *granuloma model. In this model, murine bone marrow-derived macrophages (BMDM) are cultured in a chemically-defined, serum-free medium as 3-dimensional spheroids in agarose wells. Cells maintain viability and macrophage phenotypic characteristics when exposed to sub-lethal doses of particulates up to14 days. Validation of this model for granuloma formation was done using carbon black particles (Printex 90) and crocidolite asbestos fibers as negative and positive reference materials, respectively, as recommended by an ILSI Working Group [19]. Three commercial types of MWCNTs were tested for their ability to induce granuloma formation using this *in vitro *3D model. Induction of granuloma formation was evaluated by formation of stable cellular aggregates, analysis of morphological changes characteristic of epithelioid macrophages, and activation phenotype of macrophages in response to high aspect ratio nanomaterials.

## Results

### Characterization of reference and sample materials

**Figure 1:**
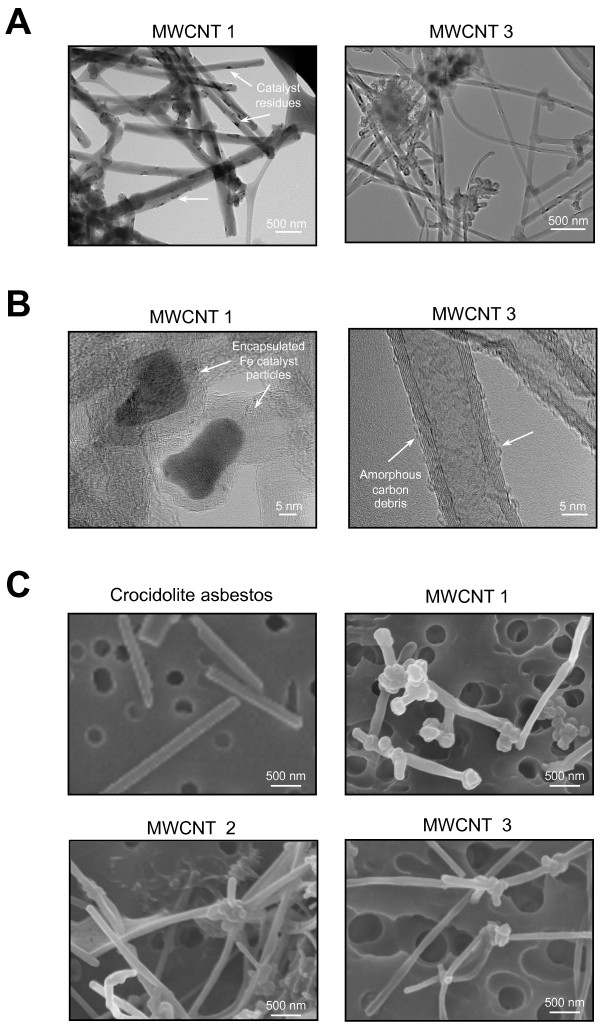
**Transmission and scanning electron microscopy images of test materials**. **A-B) **Geometry and surface features of MWCNTs. SEM imaging **(A) **shows individual carbon nanotubes. MWCNT 1 shows encapsulated metal catalyst residues and MWCNT 3 shows amorphous surface carbon residues as imaged using HR-TEM **(B)**. Both samples have the characteristic crystalline structure of carbon nanotubes. MWCNT geometry shows rigid tubes, iron catalyst residues embedded on the surface and encapsulated within well-ordered walls and surface with amorphous carbon debris. Analysis by TEM and HR-TEM. **C) **Crocidolite asbestos fibers are individually dispersed compared to cross-linking of MWCNTs into a 3D structure as shown by SEM imaging of native dry materials.

**Figure 2:**
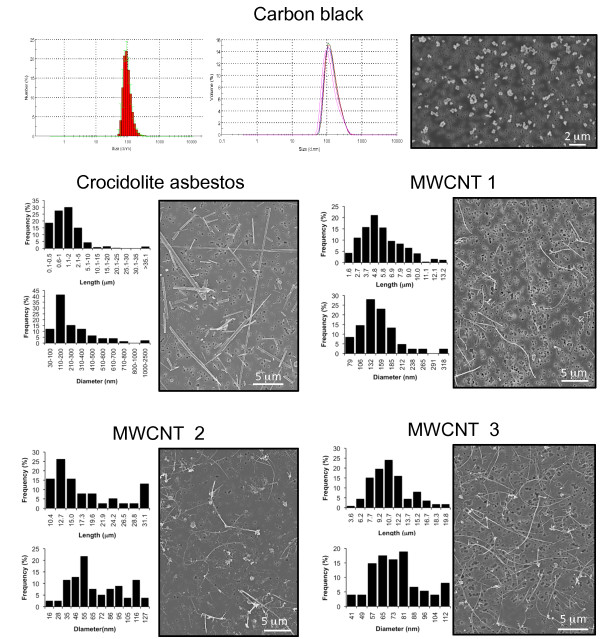
**Size distribution in serum-free medium**. Materials were dispersed in serum-free medium and collected onto a filter prior to cellular exposure shown by SEM imaging.

Materials were characterized as summarized in Table 1. All materials were evaluated and used "as produced" without further purification or modification. Transmission electron microscopic (TEM) images illustrate the overall geometry of MWCNTs (Figure 1A). High-resolution transmission electron micrographs (HR-TEM) show the wall thickness and hollow core width of the MWCNTs as well as the presence of amorphous carbon on the tube outer surfaces and carbon-encapsulated iron catalyst particles (Figure 1B). In some cases the nanotubes appear to be locally fused with or attached to neighboring tubes to form a higher order superstructure or complex 3D aggregate (Figure 1C). When suspended in serum-free medium, most of the MWCNTs are present as individual tubes or small aggregates indicating adequate dispersion for delivery to macrophages (Figure 2).

**Table 1:** Summary of material characterization

Material	Length (μm)	Diameter (nm)	Surface area (m^2^/g)	Fe content (wt-%)
Carbon black		141 ± 0.9^(1)^	270^(3)^	0.032^(5)^
Asbestos	2.8 ± 2.6 ^(1)(2)^	116 ± 112^(1)(2)^	9.1^(2)^	22.2^(2)^
MWCNT 1	6.1 ± 2.5^(1)^	163 ± 49^(1)^	10^(3)^	< 0.1^(5)^
MWCNT 2	18.8 ± 7.4^(1)^	69 ± 30^(1)^	200^(3)^	1.8^(5)^
MWCNT 3	11.7 ± 3.6^(1)(4)^	80 ± 18^(1)(4) ^	20^(3)^	0.2^(5)^

^(1) ^Size distribution in serum-free medium was evaluated by SEM and TEM using NIH Image J software. Mean ± SD. Particle size distribution of carbon black was evaluated by DLS.

^(2) ^As reported in Moalli *et al*., 1987 [31] and in Guo *et al*., 2007 [37].

^(3) ^Determined by nitrogen vapor adsorption using BET theory.

^(4) ^As reported in Poland *et al*., 2008 [35].

^(5) ^Determined by inductively coupled plasma atomic emission spectroscopy (ICP-AES).

### Determination of sub-lethal dose

**Figure 3:**
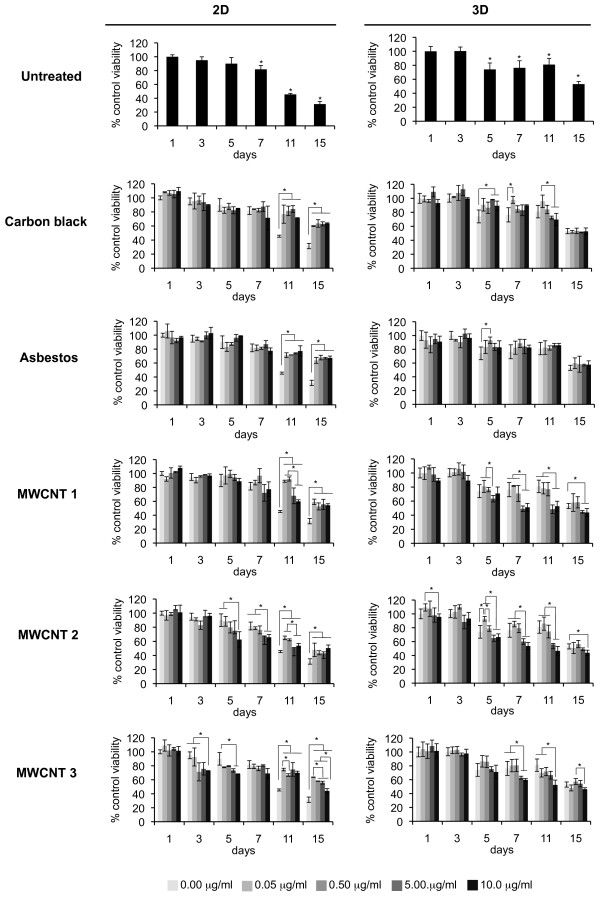
**Macrophage survival in 2D and 3D cultures**. 2D and 3D BMDM cultures were exposed to 0.05 μg/ml (0.038 μg/cm^2^), 0.5 μg/ml (0.38 μg/cm^2^), 5 μg/ml (3.8 μg/cm^2^) and 10 μg/ml (7.6 μg/cm^2^) of carbon black, asbestos fibers or MWCNTs. Total DNA content was used as a measure of cell number. Bars represent the mean ± SD of three separate determinations, *p < 0.05.

Doses with minimal effects on cell viability over 14 days following exposure to reference materials or MWCNTs (Figure 3) at 0.05 μg/ml (0.038 μg/cm^2^), 0.5 μg/ml (0.38 μg/cm^2^), 5 μg/ml (3.8 μg/cm^2^) or 10 μg/ml (7.6 μg/cm^2^) were selected. Total DNA content was used to monitor cell number as described in Materials and Methods. Cells in 2D cultures showed a significantly greater loss in viability after 7 days compared to 3D cultures. Cytotoxicity induced by particulates varies with dose and time with significant loss of cells at doses of 5 and 10 μg/ml after 3 and 5 days (Figure 3). Sub-lethal doses of 0.05 and 0.5 μg/ml were selected to evaluate induction of granuloma formation after 3-14 days in serum-free cultures of nonadherent macrophages.

### Potential of high aspect ratio nanoparticles to induce granulomas

**Figure 4:**
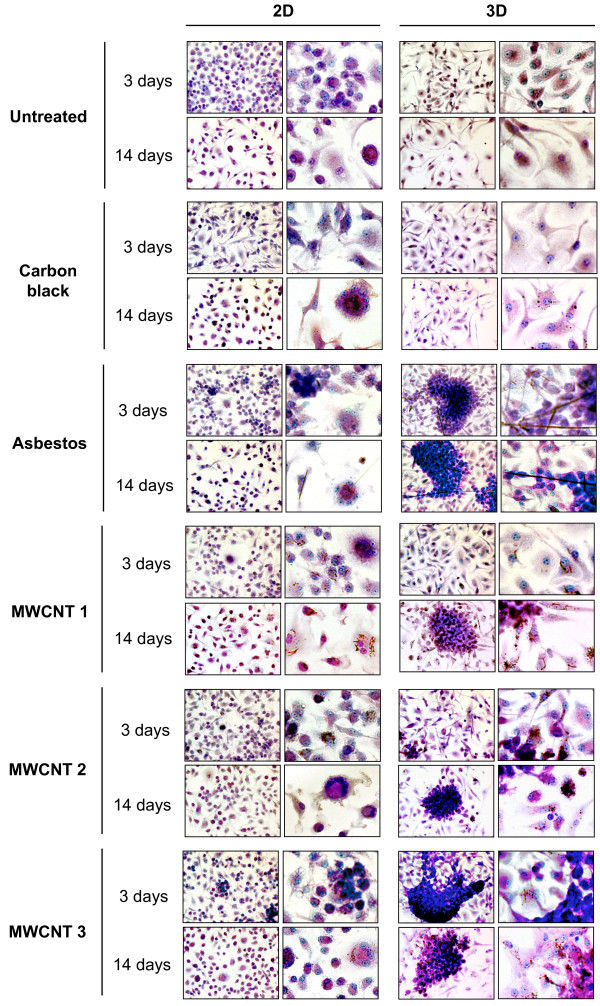
**Light microscopic morphology and kinetics of macrophage aggregation in 2D and 3D cultures**. BMDM were exposed to 0.5 μg/ml (0.38 μg/cm^2^) of particulates. Formation of stable cellular aggregates was evaluated at 3 and 14 days post-exposure. Macrophages were stained with May-Grünwald-Giemsa as described in Materials and Methods.

Formation of stable cellular aggregates in 2D and 3D cultures was evaluated at 3, 7, 10 and 14 days after exposure to 0.05 or 0.5 μg/ml of particulates (Figure 4 and Additional file 1). Following 3D culture, cells were transferred into tissue-culture-treated glass wells where cells disaggregate, attach, and spread or remain as a stable aggregate. Granuloma formation was defined as the presence of stable, cohesive 3D cellular aggregates 24 hrs after re-plating. Cells were fixed, stained with May-Grünwald-Giemsa stain, and visualized by light microscopy.

In 3D cultures, formation of cellular aggregates in response to asbestos fibers or MWCNT 1 and 2 was found to be dose-dependent. When treated at 0.05 μg/ml, macrophages form aggregates after 10 days (Additional file 1). When exposed to 0.5 μg/ml of asbestos fibers, cellular aggregates form after 3 days (Figure 4) and MWCNT 1 and 2 induced stable aggregates after 7 days (Additional file 1). Exposure to 0.05 or 0.5 μg/ml of MWCNT 3 induced aggregation after 3 days (Additional file 1). Exposure to carbon black did not induce cellular aggregation at any dose or time. In 2D cultures, untreated cells or cells exposed to 0.05 μg/ml (data not shown) or 0.5 μg/ml (Figure 4) of particulates showed formation of multinucleated giant cells. This effect may be due to prolonged attachment to a non-degradable surface in the presence of serum proteins as described previously [20,21].

### Morphological characteristics of *in vitro *granulomas

**Figure 5:**
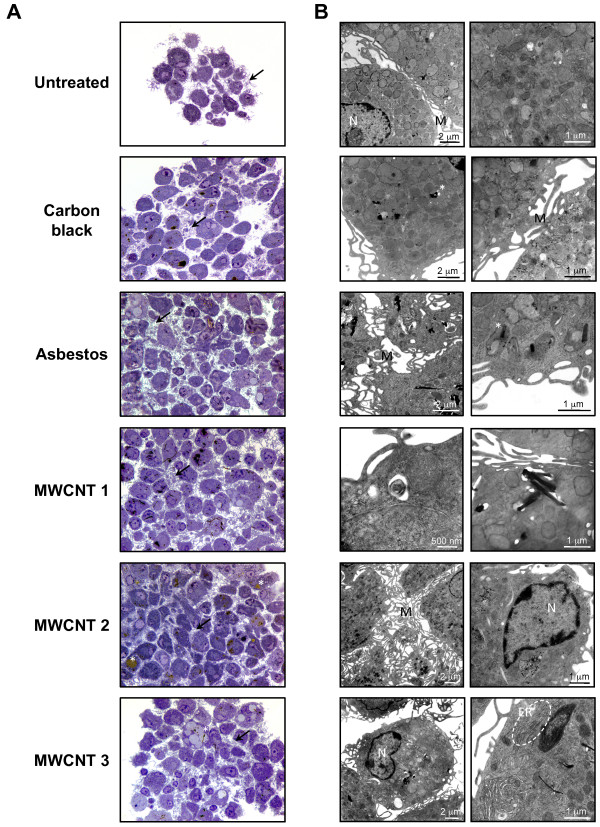
**Morphology of epithelioid granulomas after 14 days of exposure to asbestos fibers or MWCNTs in 3D cultures**. After 14 days, cells were fixed *in situ*, embedded in plastic, and sectioned. 0.5 μm sections were stained with toluidine blue **(A) **for light microscopy. Magnification: 1000×. Ultrathin sections were stained with uranyl-acetate/lead citrate for transmission electron microscopy **(B)**. Untreated cells or cells exposed to 0.5 μg/ml (0.38 μg/cm^2^) of carbon black have short surface microvilli and well-preserved cellular organelles. Exposure to 0.5 μg/ml (0.38 μg/cm^2^) of asbestos fibers or commercial MWCNTs induces epithelioid granulomas characterized by compact cell aggregates with prominent interdigitating surface microvilli. Asbestos fibers or MWCNTs are usually localized in cytoplasmic vacuoles and cellular organelles are well preserved. Black arrow and M: microvilli, N: nucleus, *: vacuole containing particulates, ER: endoplasmic reticulum.

To evaluate whether the aggregates induced by high-aspect ratio particulates show morphological features of epithelioid granulomas, cellular morphology was evaluated after 14 days of exposure to reference and test materials. Bone marrow-derived macrophages in 3D cultures were exposed to 0.5 μg/ml of particulates for 14 days, fixed *in situ*, embedded in plastic, and sectioned at 0.5 μm for light microscopy (Figure 5A) and at 90 nm for transmission electron microscopy (Figure 5B).

Untreated cells and cells exposed to carbon black particles show abundant cytoplasm, nuclei with an open chromatin pattern, prominent nucleoli, intact mitochondria, and short surface microvilli (Figure 5A and 5B). In contrast, asbestos fibers or MWCNTs induced well-formed, compact macrophage aggregates with ultrastructural features of epithelioid macrophages (Figure 5A). Most prominent is the extensive interdigitation of elongated, arborized surface microvilli. Particulates are contained in cytoplasmic membrane-bound vesicles with occasional free particulates observed in the cytoplasm (Figure 5B). Epithelioid macrophages contain abundant endoplasmic reticulum and large cytoplasmic vacuoles (Figure 5B) that are also characteristic of granulomas [22,23].

### Phagocytosis of high aspect ratio nanomaterials by macrophages in 3D cultures

**Figure 6:**
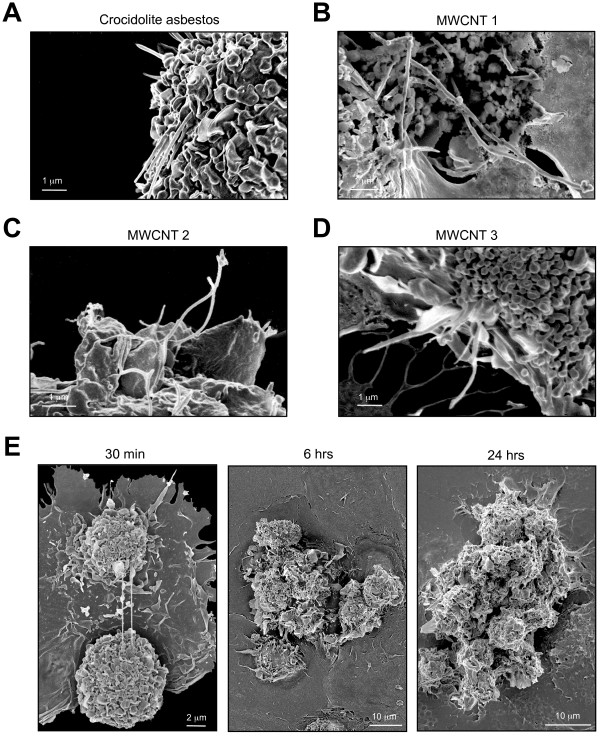
**Phagocytosis of asbestos fibers and MWCNTs and cell aggregation in 3D cultures**. **A-D) **Evidence of frustrated phagocytosis after 6 hrs of exposure to 1.25 μg/ml of asbestos fibers or MWCNTs. At the times specified, 3D cultures were transferred to a glass-coverslip, incubated for 15 min to allow re-attachment and fixed for examination by scanning electron microscopy. **A) **Crocidolite asbestos fibers. **B) **MWCNT 1. **C) **MWCNT 2. **D) **MWCNT 3. **E) **Time course of cell aggregation after exposure to 1.5 μg/ml of MWCNT 1 in 3D cultures.

To evaluate whether macrophages in 3D cultures internalize MWCNTs similar to asbestos fibers, cells were exposed to 1.25 μg/ml of asbestos or MWCNTs for 6 hours and evaluated by scanning electron microscopy (SEM) (Figure 6). Macrophages in 3D culture recognize and attempt to internalize asbestos fibers and MWCNTs. Both asbestos fibers and MWCNTs show evidence of incomplete phagocytosis after 6 hrs of exposure (Figure 6A-D). After 30 min of exposure to MWCNTs, cells attach to long MWCNTs followed by progressive cell aggregation after 6-24 hours (Figure 6E). Although the onset of MWCNT internalization occurs early, stable cohesive cell aggregates are not established until 3 days after exposure to asbestos fibers or MWCNT 3 or 7 days after exposure to MWCNT 1 and 2 (Additional file 1).

### Cellular activation in 3D cultures in response to particulates

Following exposure to microorganisms, macrophages undergo classical activation (M1 or pro-inflammatory phenotype) or differentiation towards alternatively activated macrophages (M2 or pro-fibrotic phenotype) [24-26]. Prototypical markers of the M1 phenotype include proinflammatory mediators including TNF-α, IL-1β, and IL-6 [27], and up-regulated iNOS expression [28]. The anti-inflammatory or profibrotic M2 phenotype is characterized by induction of arginase-1 (Arg-1) and increased synthesis and release of mannose receptor (MR/CD206) [26].

First, we determined the ability of 2D and 3D cultures of macrophages to express a pro- or anti-inflammatory response by exposure to 100 ng/ml of lipopolysaccharide (LPS) or 20 ng/ml of IL-4 and IL-13, respectively (Additional file 2). LPS induced TNF-α release, iNOS expression, and decreased MR expression (Additional file 2 Figure S1). IL-4 or IL-13 induced Arg-1 expression, as well as expression and release of both MR and YM-1 (Additional file 2 Figure S2).

Although the ability of macrophages to respond towards a pro- or anti-inflammatory stimulus under 2D or 3D conditions is not impaired, we were unable to detect significant differences in the levels of these inflammatory mediators using ELISA (TNF-α) or Western blot assays (iNOS and Arg-1) when macrophages were exposed to 0.5 μg/ml of particulates compared to untreated cells. Additionally, no significant differences in IL-1β release were detected following exposure to these reference and test particulate materials at the sublethal doses used in these experiments in the absence of priming by LPS or phorbol ester (Additional file 3).

**Figure 7:**
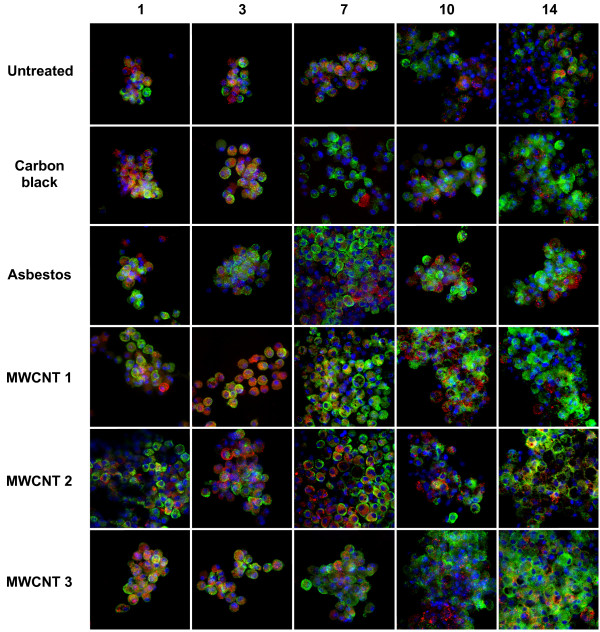
**Early induction of TNF-α expression followed by late induction of mannose receptor (MR) in 3D cultures**. Cells were exposed to 0.5 μg/ml (0.38 μg/cm^2^) of particulates. After 1, 3, 7, 10 and 14 days, cells were incubated with Brefeldin A for 6 hrs, fixed *in situ *and co-immunostained for TNF-α (red), MR (green), and counterstained with DAPI (blue nuclear fluorescence). A three-day exposure to asbestos or MWCNTs resulted in an increase in TNF-α expression. Prolonged exposure to all nanomaterials resulted in an increase in MR expression. Samples were analyzed by confocal microscopy using the appropriate filters. Photographs are representative of Z-stacks of three individual samples. Magnification: 400×.

**Figure 8:**
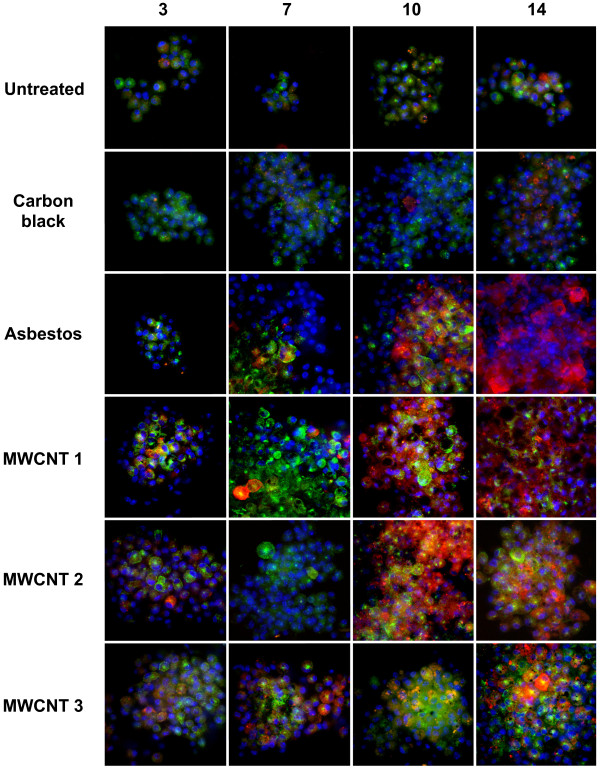
**Induction of iNOS followed by arginase-1 (Arg-1) expression in 3D cultures**. Cells were exposed to 0.5 μg/ml (0.38 μg/cm^2^) of particulates. After 3, 7, 10 and 14 days, cells were fixed and co-immunostained for iNOS (green), Arg-1 (red), and counterstained with DAPI. Cells exposed to asbestos and MWCNT 1 and 3 showed earlier co-expression of iNOS and Arg-1. The increasing levels of expression of these two mediators indicate progression towards a profibrotic response. Samples were analyzed by confocal microscopy using the appropriate filters. Photographs are representative of Z-stacks of three individual samples. Magnification: 400×.

To evaluate whether high aspect ratio nanomaterials induce markers of M1 (iNOS, TNF-α) or M2 activation (Arg-1, MR), the kinetics of macrophage activation induced in 3D cultures following exposure to 0.5 μg/ml of particulates were determined *in situ *using dual immunofluorescence imaging and confocal microscopy (Figure 7 and 8).

Compared to untreated cells, exposure to asbestos or MWCNTs induced an early increase in TNF-α expression and decreased expression of MR between 1 and 3 days. After day 7, this activation phenotype changes with variable TNF-α expression and increased MR expression following exposure to all particulates (Figure 7).

Exposure to asbestos or MWCNTs in 3D cultures also induces early iNOS expression after 3 - 7 days, followed by a progressive increase in Arg-1 expression (Figure 8). Induction of both iNOS and Arg-1 in untreated cells or cells exposed to carbon black was also evident at later time points but to a lesser extent than asbestos or MWCNT-treated cells (Figure 8). Phenotypic heterogeneity is evident following all exposures. The kinetics of expression of different phenotypic markers is not identical for all markers evaluated: TNF-α expression is upregulated prior to increased MR expression and no co-localization of these markers is observed (Figure 7). In contrast, iNOS expression increases early and is prolonged with a gradual transition to increasing Arg-1 co-expression between 7-14 days. Untreated cells or cells exposed to carbon black showed low levels of Arg-1 expression (Figure 8). This phenotypic heterogeneity and co-expression of M1 and M2 markers by a subset of macrophages following exposure to asbestos or MWCNTs is similar to the complex phenotype shown by murine macrophages isolated from healing wounds [29].

### Particulate agglomeration before and after exposure

**Figure 9:**
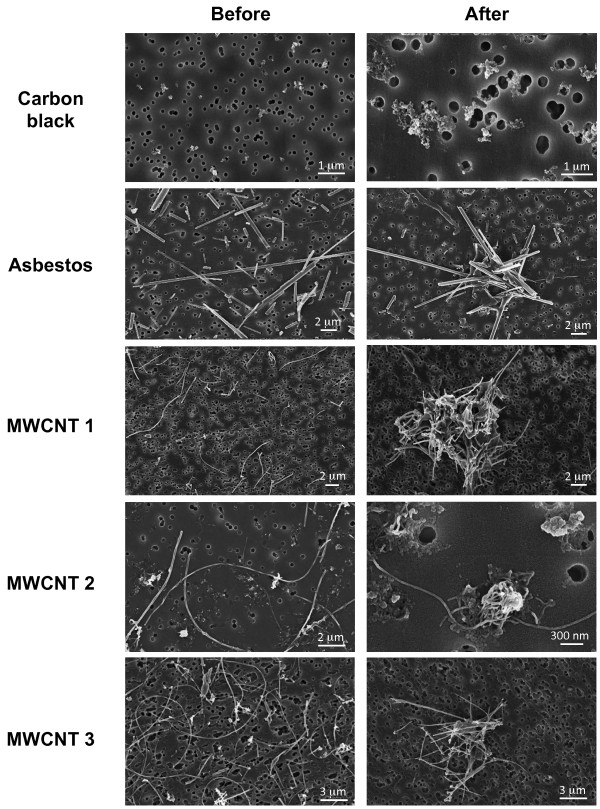
**Agglomeration state of particulates before and after exposure**. Dispersion of reference and experimental materials in serum-free medium prior to macrophage exposure was evaluated at 5 μg/ml for particles, at 2.5 μg/ml for asbestos fibers, MWCNT 2 and 3, and at 0.5 μg/ml for MWCNT 1. After 14 days of exposure in serum-free medium, 3D cultures were lysed and particulates were recovered and imaged by SEM as described in Materials and Methods. Limited agglomeration of carbon black particles is evident in comparison with larger agglomerates of asbestos and MWCNTs post-exposure.

Dispersion of reference and test particulates in serum-free medium was compared using scanning electron microscopy (SEM) before and after exposure. Particle suspensions of carbon black were evaluated at 5 μg/ml; asbestos fibers, MWCNT 2 and 3 were evaluated at 2.5 μg/ml, and MWCNT 1 at 0.5 μg/ml as described in Materials and Methods. Prior to exposure, single particles and well-dispersed asbestos fibers and carbon nanotubes are evident with minimal aggregation. Following exposure to 0.5 μg/ml for 14 days in 3D cultures, limited agglomeration of carbon black particles is evident in comparison with submicron and micron-sized agglomerates of asbestos fibers and MWCNTs (Figure 9).

## Discussion

This *in vitro *model of granuloma formation uses murine BMDM differentiated *in vitro *using macrophage colony stimulation factor (M-CSF) (Additional file 4 Table S1) The phenotype of these differentiated cells resembles mature circulating blood monocytes (Additional file 4 Figure S1). These cells are recruited into the lungs [30] or the peritoneal cavity [31] in response to particulate stimuli following an early influx of neutrophils. If the particulate stimulus is toxic and biopersistent, these elicited macrophages may further differentiate into epithelioid macrophages and form a granuloma [22,23]. Activation of BMDM and formation of stable aggregates with the morphological features of a foreign body or non-immune granuloma are successfully reproduced in this serum-free, *in vitro *model only in response to high aspect ratio nanomaterials, not to spherical carbon black particles that have chemically similar surfaces, also composed of sp^2^-hybridized (graphenic) carbon.

The relative ranking of these materials for induction of granulomas in this 3D models is: MWCNT 3 > asbestos > MWCNT 1, MWCNT 2 > carbon back. Surface area is not a major determinant of granuloma formation in this data set because the MWCNT 2 with a surface area of 200 m^2^/g did induce granuloma formation while carbon black nanoparticles with a surface area of 300 m^2^/g did not.

**Figure 10:**
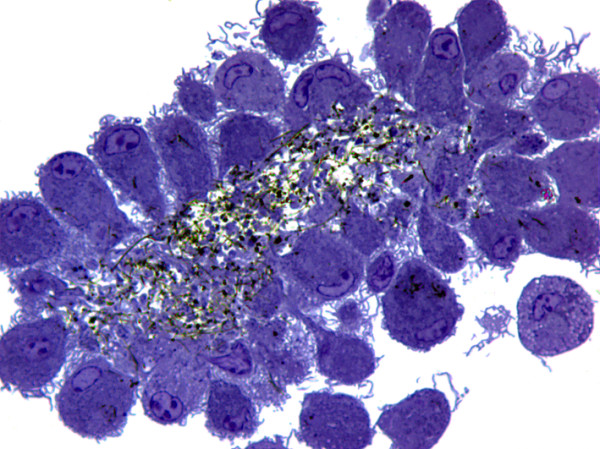
**Morphology of granuloma illustrating secondary agglomeration of carbon nanotubes by bone marrow-derived macrophages exposed initially to well-dispersed materials**. After 14 days, cells were fixed *in situ*, embedded in plastic, and sectioned at 0.5 μm, and stained with toluidine blue for light microscopy. Magnification: 1000×.

Epithelioid granulomas are a prominent feature of the lung pathology induced in the first intratracheal instillation studies using carbon nanotubes [4,32]. Initially, this lesion was interpreted as an artifact due to bolus instillation of agglomerated carbon nanotubes. Later studies using pharyngeal aspiration of SWCNTs also induced scattered foci of granulomatous inflammation as well as fibrosis [33]. This *in vitro *model of granuloma formation used well-dispersed samples of asbestos fibers and three commercial types of MWCNTs as illustrated in Figure 2. Within 3-7 days after exposure, macrophages in 3D culture formed stable aggregates with the morphological features of an epithelioid granuloma. These pathological lesions (Figure 10) are morphologically identical to granulomas induced in mice following pharyngeal aspiration of MWCNTs [11] that are identical to sample 3 used in this *in vitro *model. In this *in vivo *study, the MWCNTs were well-dispersed in synthetic lung lining fluid [34]. Ma-Hock *et al*. [10] also delivered well-dispersed MWCNT to rats by inhalation and induced multifocal granulomas, even at a dose of 0.1 mg/m^3 ^for 13 weeks.

During *in vitro *exposure in this 3D model, all of the nanomaterials underwent agglomeration (Figure 9), especially MWCNT 3 as illustrated in Figure 10. This phenomenon may be driven by several events leading to entangled fibrous agglomerates that persist after the cells are lysed. First, internalization of materials may lead to intracellular aggregation during phagocytosis. Second, over time, macrophages may undergo repeated cycles of phagocytosis and cell death, resulting in extracellular release and accumulation of agglomerated fibrous nanomaterials.

High aspect ratio nanoparticles also induce frustrated phagocytosis and formation of multinucleated giant cells similar to the response of macrophages to asbestos fibers or carbon nanotubes following pharyngeal aspiration [11] or intraperitoneal or pleural injection [35,36]. Similar to the study by Poland *et al*. [35], this *in vitro *model used a range of commercial MWCNT samples with an iron content of < 0.1 - 1.8 wt-%; however, these samples induced granulomas to the same extent as crocidolite asbestos, the positive reference sample, with an iron content of 22%. Porter *et al*. [11] used the same MWCNT as our sample 3 and did not detect generation of hydroxyl radical using electron spin resonance (ESR). Although redox activity of commercial carbon nanotubes is lower than that of amphibole asbestos fibers [37], this does not rule out a role for secondary generation of ROS by macrophages during phagocytosis of high aspect ratio nanomaterials [13].

The phenotype of BMDM induced by exposure of 3D cultures to asbestos fibers or MWCNTs also recapitulates the proinflammatory response of macrophages following inhalation of asbestos fibers. For example, inhalation of chrysotile or crocidolite asbestos by rats increased formation of nitrotyrosine in alveolar macrophages and other target cells of the lungs and pleura [38] suggesting induction of iNOS similar to this *in vitro *model. Asbestos fibers delivered by inhalation to the lungs [39] or by intraperitoneal injection to the mesothelium [31] elicit an acute inflammatory response due to the proinflammatory mediators produced by resident macrophages [19]. Intratracheal instillation of asbestos fibers or MWCNTs in rats stimulated TNF-α release relative to ultrafine carbon black particles [4]. In this *in vitro *model, asbestos fibers and MWCNTs also upregulated TNF-α protein expression in comparison to carbon black three days after exposure (Figure 7).

The proinflammatory mediators TNF-α, IL-1β, and iNOS have also been observed during the formation of immune granulomas *in vivo *[40] and *in vitro *[41]. Less is known about the mediators involved in production and healing of non-immune granulomas. Non-immune or foreign body granulomas in the lung may progress to local fibrosis [42] similar to the reaction to well-dispersed MWCNTs delivered to the lungs of rodents by pharyngeal aspiration [5]. The phenotype of activated macrophages in non-immune granulomas has not been characterized; however, arginase-1, a marker of M2 macrophage activation, is induced after 10-14 days following exposure to asbestos or MWCNTs in this *in vitro *model (Figure 8). Induction of arginase-1 is associated with a profibrotic phenotype [43] and may be useful as a biomarker to predict potential fibrosis induced by high aspect ratio nanoparticles. Co-expression of proinflammatory as well as profibrotic phenotypic markers by epithelioid macrophages may be responsible for sustained inflammation and rapid development of fibrosis induced by MWCNTs in the lungs of rodents [4,11].

The biological mechanisms responsible for formation of stable aggregates of macrophages elicited from circulating blood monocytes in non-immune granulomas are unknown [44]. Differentiation of monocytes into epithelioid macrophages is a morphological feature of granulomas. Epithelioid cells are large cells characterized by abundant endoplasmic reticulum, mitochondria, lysosomes, and cytoplasmic vesicles and vacuoles [22]. Interdigititation of long, surface microvilli may be responsible for stable adhesion of epithelioid cells and they are the ultrastructural hallmark of granulomas [23]; these morphological features are produced by exposure to asbestos fibers or MWCNTs, but not to carbon black (Figure 4). The complex 3D aggregate structure (Figure 1C) characteristic of these commercial carbon nanotube samples may promote macrophage aggregation and granuloma formation *in vitro *and *in vivo*.

A major caveat in *in vitro *nanotoxicology assays is the high dose delivered to target cells that may not reflect human exposure under realistic conditions [45]. In this *in vitro *model, 1.4 × 10^5 ^BMDM were exposed to 0.05-0.5 μg of test particulates. Elder *et al*. [46] established a subchronic NOAEL for Printex 90, the carbon black sample used in this *in vitro *study, and determined that approximately 0.1 mg was deposited in the lungs of rats after 13 weeks inhalation at a dose of 1 mg/m^3^. Assuming 3 × 10^7 ^alveolar macrophages per rat [47,48], the dose used in this *in vitro *assay is close to the deposited dose of carbon black under conditions that do not induce impaired clearance or inflammation. For comparison, airborne dust levels of MWCNTs measured by Han *et al*. [49] in a research facility were 400 μg/m^3 ^and this dose corresponded to the doses delivered to mice by pharyngeal aspiration that produced lung granulomas and diffuse interstitial fibrosis [11].

## Conclusion

The distinctive morphological and phenotypic responses of BMDM to reference particulates and MWCNTs in serum-free 3D culture establish proof-of-principle for this *in vitro *model as a potential alternative to both traditional 2D monolayer cultures and to rodent bioassays for granuloma formation. This *in vitro *3D model is a promising platform for testing of various commercial varieties of carbon nanotubes as well as other new high aspect ratio or one-dimensional nanomaterials including metallic nanowires [36], and ZnO or TiO_2 _nanorods and nanobelts [50]

## Materials and methods

### Isolation and culture of bone marrow-derived macrophages

This protocol was approved by the Brown University Institutional Animal Care and Use Committee in accordance with the Animal Welfare Act and the National Institute of Health Guide for the Care and Use of Laboratory Animals. Murine bone marrow-derived macrophages were differentiated from bone marrow cells isolated from 6- to 8- week-old C57BL/6J male mice (Jackson Laboratories, Bar Harbor, ME). Briefly, animals were sacrificed by CO_2 _asphyxiation, femurs were removed, and bone marrow was harvested by flushing with 3 ml of Dulbecco's Modified Eagle medium (DMEM; Invitrogen, Carlsbad, CA) supplemented with 20% fetal bovine serum (FBS; Atlanta Biologicals, Lawrenceville, GA), 10 units/ml penicillin, and 10 μg/ml of streptomycin (Invitrogen, Carlsbad, CA). Cells were collected in a 50 ml conical tube on ice, centrifuged at 500 g for 5 min at 4°C. Cells were resuspended in fresh media supplemented with 20 ng/ml of M-CSF (R&D Systems, Minneapolis, MA) and incubated overnight at 37°C in a humidified atmosphere of 5% CO_2_. Non-adherent cells were harvested and transferred to 10 cm non-tissue culture-treated polystyrene dishes. After 3 days the medium was replaced with fresh medium and, on day 7, non-adherent cells were removed. Adherent cells were washed 5 times with PBS, and 10 ml of serum-free medium (SFM) was added to each dish: X-Vivo 15 medium supplemented with 1X HL-1 (Lonza Rockland Inc., Rockland, ME), 20 ng/ml M-CSF and 10 units/ml penicillin and 10 μg/ml of streptomycin. On day 10, cells were detached with 5 mM of EDTA, centrifuged at 500 g for 5 min at 4°C, resuspended, and plated in SFM at 0.14 × 10^6 ^cells/ml. A 200 μl volume of cell suspension (35,000 cells) was added to each well of a 96-well microplate. For three dimensional cultures, wells were previously coated with 50 μl of 3% (wt/vol) low melting point agarose (SeamPlaque, Lonza Rockland Inc.) dissolved by boiling in endotoxin-free water (Sigma-Aldrich, St. Louis, MO). Coated wells were equilibrated with SFM for 24 hrs at 37°C and 5% CO_2 _in humidified chamber prior to use. In order to avoid endotoxin contamination, glassware and plastic containers were washed with PyroCLEAN™ detergent (AlerCHEK, Portland, ME) and autoclaved before use. Cells were kept up to 14 days and 2/5 of medium was replaced with fresh medium every other day. Light microscopy did not reveal any contamination with fibroblasts after 14 days.

### Materials and cell treatments

Crocidolite asbestos fiber stocks ranging from 0.1 to 35 μm in length and 30 to 3000 nm in diameter, originally characterized by the International Union Against Cancer (UICC, Timbrell, 1971/72), were purchased from Duke Scientific (Palo Alto, CA). Carbon black particles (Printex-90, Evonik, Essen, Germany) with an average primary particle size of 14 nm and MWCNT 3 produced by chemical vapor deposition (CVD) with average dimension of 13 μm in length and 85 nm in diameter (MWCNT 3, MWCNT-7, Mitsui & Co. Ltd; Tsukuba, Ibaraki, Japan) were a gift of Dr. Günter Oberdörster (University of Rochester, Rochester, NY). MWCNT 1 (mean length 7 ± 2 μm, mean diameter 140 ± 30 nm) and MWCNT 2 (mean length 30 μm, mean diameter 35 ± 10 nm) produced by CVD were purchased from MER Corporation (Tucson, AZ). Size distribution of MWCNTs in serum-free medium was evaluated by SEM and TEM using NIH Image/ImageJ software (Table 1).

Asbestos fibers were baked in an oven at 250°C for 16 hrs to remove endotoxin. Stocks at 0.5 mg/ml in endotoxin free water (Sigma) were autoclaved and stored at 4°C. This sterilization protocol did not decrease the biological activity of asbestos fibers following intraperitoneal injection in mice [31] or redox activity as assessed by plasmid DNA break assay [37]. Removal of endotoxin and sterilization of carbon nanomaterials were done by heating at 400°C for 30 min in a furnace under continuous flow of nitrogen gas to prevent oxidation.

Stocks at 0.5 mg/ml in sterile endotoxin free water were stored at 4°C. Working solutions were prepared by dilution of stock suspensions in SFM. Briefly, stock suspensions were water-bath sonicated for a minimum of 30 min using a tabletop ultrasonic water bath (Branson Ultrasonic Corporation, Danbury, CT). After 30 min, the sample was mixed by pipeting up and down during sonication and 1 ml of the stock was transferred to a clean tube using wide bore, low retention tips (Axygen INC, Union City, CA), then brought to the desired concentration with SFM and sonicated for an additional 5 minutes. A volume of 50 μl of working solution was added to each well at a final volume of 250 μl. Dry materials and suspensions were handled under a Class IIB laminar flow hood equipped with Hepa filters with external exhaust. Endotoxin levels of the stock suspensions were less than 0.24 EU/ml as determined by gel-clot assay (Embryotch™ Laboratories, Wilmington, MA).

### Determination of cell viability

Total DNA content was used as an indicator of total cell number. At the specified time, culture medium was removed and cells were digested with fungal proteinase K (Invitrogen) at a final concentration of 50 μg/ml (≥ 1 unit/ml) in TE buffer (200 mM Tris-Cl, 20 mM EDTA, pH 7.5) overnight at 37°C and stored at -20°C until use. Digested cell suspensions were brought to 300 μl of final volume with TE buffer and a 20 μl aliquot was used to determine DNA content. Double-stranded DNA was quantified using Quant-it Picogreen dsDNA kit (Molecular Probes, Eugene, OR) following the manufacturer's protocol for a 200 μl reaction assay. Briefly, samples were diluted with TE buffer to a final volume of 100 μl and 100 μl of the Quant-it Picogreen working solution was added to each sample, mixed, and incubated for 5 min at room temperature protected from light. Samples were excited at 480 nm and fluorescence emission intensity was measured at 520 nm using a fluorescence microplate reader (SpectraMax M2, Molecular Devices, Sunnyvale, CA). Under these conditions, the linear detection range of the assay was used for all treatments and no interference due to test particulates was observed.

### Indirect immunofluorescence

For intracellular staining of TNF-α and mannose receptor (MR), cells were pre-incubated with Brefeldin A (BD Biosciences, Franklin Lakes, NJ) for 6 hrs prior to fixation. Cells were fixed in 4% paraformaldehyde in PBS for 30 min at room temperature, washed 3 times with 0.1% BSA/PBS, and permeabilized in 0.25% (w/v) saponin/blocking solution for 10 min. After washing, cells were incubated for 30 min in blocking solution. Following incubation overnight at 4°C with primary antibodies against arginase-1, iNOS, TNF-α (BD Biosciences) or MR (AbD Serotec, Oxford, UK) in blocking solution, cells were washed with PBS and incubated with secondary antibodies in blocking solution for 2 hrs at room temperature. After three washes with PBS, cells were mounted on glass slides using Vectashield (Vector Lab. Inc., Burlingame, CA) with DAPI as a nuclear counterstain. Samples were analyzed using SlideBook software (Intelligent Imagine Innovations, Denver, CO) and spinning disk confocal scanning microscopy (Olympus IX81, Olympus Corporation, Tokyo, Japan) using appropriate filters for dual wavelength immunofluorescence.

### May-Grünwald-Giemsa stain

Bone marrow-derived macrophages in agarose-coated wells were transferred to glass LabTek wells (Nunc, Thermo Fisher Scientific Inc. Rochester, NY) and incubated for 6 to 24 hrs to allow cell attachment and spreading. Attached cells were fixed with methanol pre-chilled to -20°C. Slides were immersed for 3 min in filtered May-Grünwald (Sigma) and 2 min in Giemsa (Sigma) followed by 30 sec in acetone, 2:1 acetone: xylene, 1:2 acetone: xylene and xylene. Slides were air-dried and mounted with Permount™ Mounting Media (Fisher, Pittsburgh, PA). Light micrographs were taken using a Nikon Eclipse 501 (Nikon Instruments Inc, Melville, NY) microscope. Images were acquired and analyzed with SOPT Insight FireWire imaging software (SPOT Imaging Solutions, Sterling Heights, MI).

### Toluidine blue stain

Macrophages in 3D cultures were fixed as pellets at room temperature for 30 min in 2% glutaraldehyde in 0.1 M sodium cacodylate buffer, pH 7.4, rinsed, and stored at 4°C in 0.1 M sodium cacodylate buffer with 8% sucrose for further processing. Cells were post-fixed with 1% osmium tetroxide in 0.1 M sodium cacodylate buffer for 30 min at room temperature and rinsed 3 times with buffer for 5 min. Following dehydration in graded ethanols (70%, 90% and 95%) for 10 min and 3 changes of 100% ethanol for 5 min, samples were infiltrated for 12-18 hours in a 1:1 mixture of 100% ethanol and embedding resin (Spurr Low Viscosity Embedding Medium, Electron Microscopy Sciences, Hatfield, PA). Samples were placed in 100% embedding media for 4 hours, and placed in a mold with fresh resin. Blocks were polymerized in an oven at 65°C overnight and sectioned on a Reichert Ultracut Ultramicrotome at a thickness of 0.5 μm using a diamond knife (Diatome US, Hatfield, PA). Following toluidine blue stain, slides were air dried and mounted in Permount™ Mounting Media (Fisher). Light micrographs were taken using a Nikon Eclipse 501 microscope (Nikon Instruments Inc, Melville, NY) and SOPT Insight FireWire software.

### Transmission Electron Microscopy

90 nm sections from the same plastic-embedded block used for toluidine blue stain were stained with 1% uranyl acetate in 50% methanol for 10 min and lead citrate for 5 min, placed on copper grids, and viewed with a Phillips 410 Transmission Electron Microscope equipped with an Advantage HR CCD camera (Advanced Microscopy Techniques, Danvers, MA). Images were acquired and analyzed with AMT's imaging software.

### Scanning electron microscopy (SEM)

#### Morphological analysis of materials prior to exposure

Materials were dispersed in serum-free medium at different concentrations, followed by water-bath (Branson Ultrasonic Corporation) sonication for 30 min and vacuum filtered through a PVCD membrane (Millipore, Billerica, MA).

#### Morphological analysis of materials post-exposure

After 14 days of exposure, cells from 3D cultures were collected, washed, and resuspended in lysis buffer (2% Triton X-100, 10 mM Tris-HCl, pH 8, 150 mM NaCl, 10 mM NaN_3_, 10 mM EDTA, 5 mM iodoacetamide, 2 mM phenylmethylsulfonyl fluoride, 1 g/ml pepstatin, and 1 M leupeptin). After incubation for 30 min on ice and vortexing every 10 min, cell lysates were centrifuged at 10,000 × g for 10 min at 4°C in a Sorvall Legend Mach 1.6 R centrifuge (Kendro Laboratory Products Inc, Asheville, NC). Supernatants were discarded, pellets were resuspended in 50 ml of methanol, followed by water-bath sonication for 1 hr and vacuum filtered through a PVCD membrane (Millipore).

#### Cellular phagocytosis and aggregation

After 0.5, 6, and 24 hrs of exposure to 1.25 μg/ml (1 μg/cm^2^) of asbestos fibers or MWCNTs, cells in 3D cultures were transferred to a glass coverslip, incubated for 15 min to allow attachment and fixed with 2.5% gluteraldehyde in 0.1 M cacodylate buffer.

All images were taken using a LEO 1530 Thermally-Assisted Field Emission (TFE) Scanning Electron Microscope (SEM) (LEO, Germany).

### Statistical analysis

Data were analyzed using MatLab software (MathWorks, Natick, MA). All results are presented as the mean ± standard deviation (SD). Differences were compared by Scheffé's method after analysis of variance (ANOVA). A *p *value < 0.05 was considered as statistically significant.

## Competing interests

The authors declare that they have no competing interests.

## Authors' contributions

AY and RHH characterized and imaged the synthetic reference and test materials. VCS conducted the cell culture experiments and morphological studies using light and fluorescence confocal microscopy. PW carried out ultrastructural imaging of the 3D cultures. VCS analyzed the data and prepared graphics. ABK and VCS designed the experiments. VCS prepared the manuscript with revision by ABK and RHH.

All authors have read and approved the final manuscript.

## Supplementary Material

Additional file 1 **Morphology and kinetics of macrophage aggregation in 3D cultures**. BMDM in 3D cultures were exposed to 0.05 μg/ml (0.038 μg/cm^2^) or 0.5 μg/ml (0.38 μg/cm^2^) of particulates. Formation of stable cellular aggregates was evaluated at 3, 7 and 10 days post-exposure. Macrophages were stained with May-Grünwald-Giemsa as described in Materials and Methods. Magnification: 400×. Click here for file

Additional file 2 **M1 and M2 macrophage polarization in 2D and 3D cultures**. This file contains a detailed description of the pro- and anti-inflammatory mediators induced in 2D and 3D cultures in response to LPS, IL-4 and IL-13 under serum-free conditions. Click here for file

Additional file 3 **IL-1β secretion after exposure to particulates in 3D cultures of macrophages**. After 3, 7 and 14 days of exposure to **A) **0.05 μg/ml (0.038 μg/cm^2^), **B) **0.5 μg/ml (0.38 μg/cm^2^), or **C) **2.5 μg/ml (1.9 μg/cm^2^) of particulates, secretion of IL-1 was evaluated in cell supernatants by ELISA. Bars represent the mean ± SD of triplicates. Click here for file

Additional file 4 **Optimization of cell culture conditions for long-term survival of macrophages and characterization of cellular phenotype**. This file includes the list of the cell culture media evaluated for optimal macrophage survival, comparison of cell survival between serum-free and serum-containing medium, and a phenotypic description of the cell population. Click here for file
