# Supplementary figures and images for: A 3-dimensional in vitro model of epithelioid granulomas induced by high aspect ratio nanomaterials
Source: Part Fibre Toxicol. 2011 May 18;8:17. doi: 10.1186/1743-8977-8-17 (PMC3120675; doi:10.1186/1743-8977-8-17)

**A**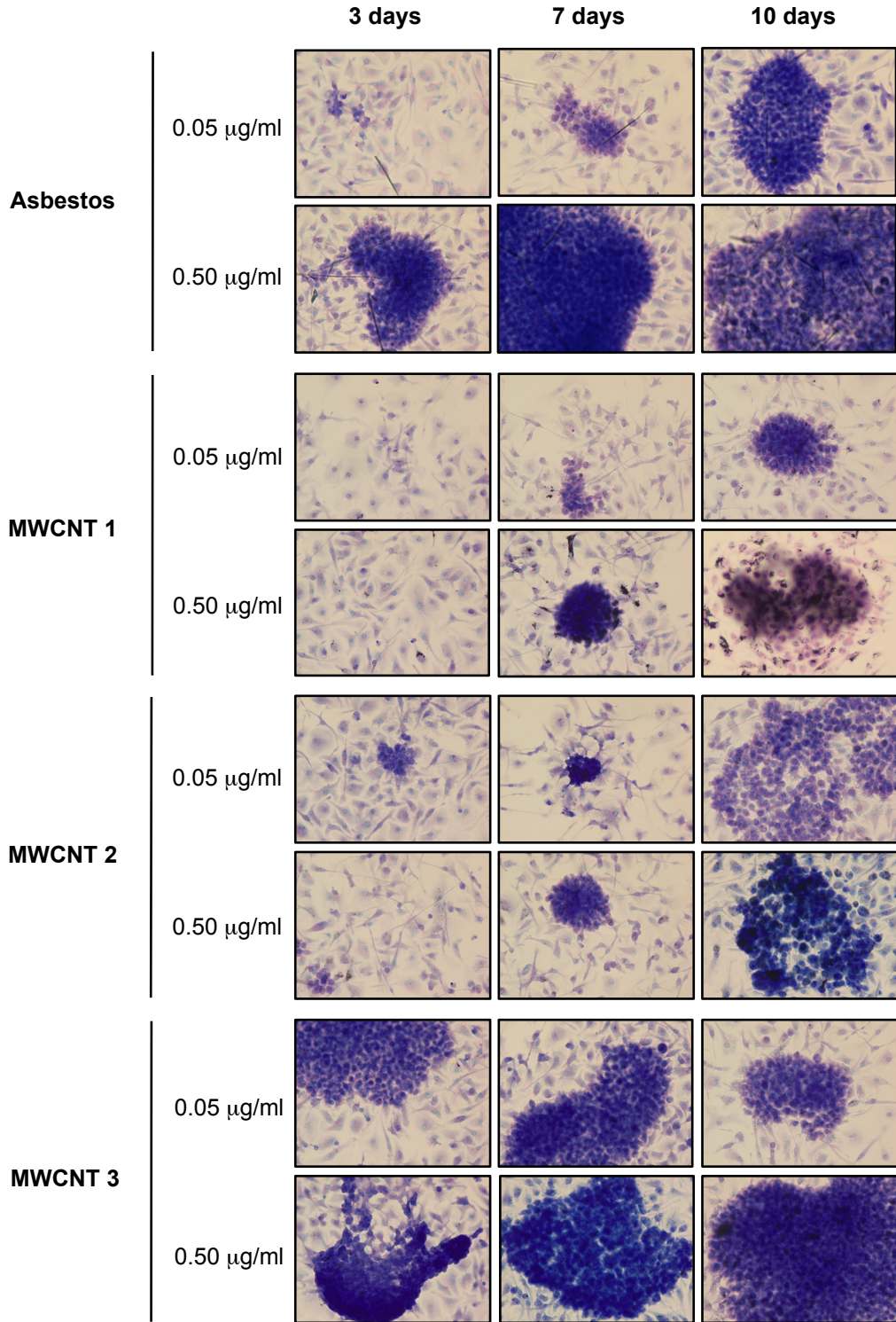**B**

|          | 0.05 $\mu\text{g/ml}$ |   |    |    | 0.50 $\mu\text{g/ml}$ |   |    |    |
|----------|-----------------------|---|----|----|-----------------------|---|----|----|
|          | 3                     | 7 | 10 | 14 | 3                     | 7 | 10 | 14 |
| Asbestos |                       |   | X  | X  | X                     | X | X  | X  |
| MWCNT 1  |                       |   | X  | X  |                       | X | X  | X  |
| MWCNT 2  |                       |   | X  | X  |                       | X | X  | X  |
| MWCNT 3  | X                     | X | X  | X  | X                     | X | X  | X  |

Supplement: Additional file 1 — Morphology and kinetics of macrophage aggregation in 3D cultures. BMDM in 3D cultures were exposed to 0.05 μg/ml (0.038 μg/cm2) or 0.5 μg/ml (0.38 μg/cm2) of particulates. Formation of stable cellular aggregates was evaluated at 3, 7 and 10 days post-exposure. Macrophages were stained with May-Grünwald-Giemsa as described in Materials and Methods. Magnification: 400×. [file 1743-8977-8-17-S1.PDF]

**A**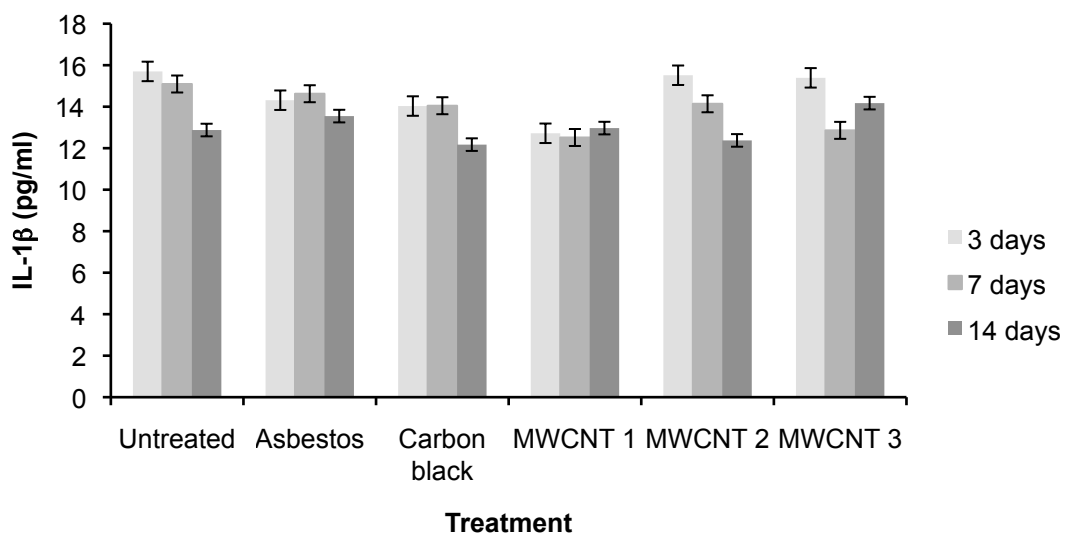**B**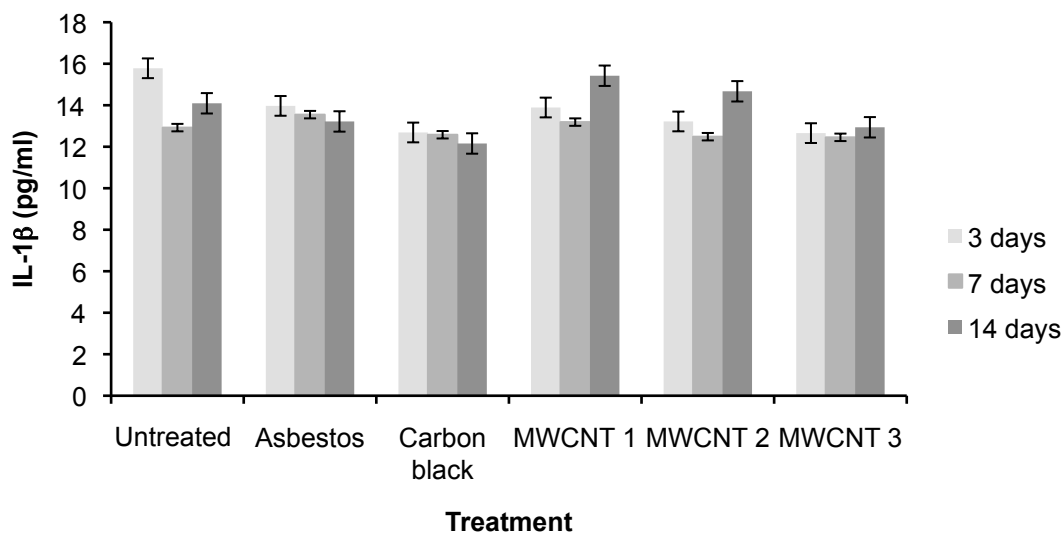**C**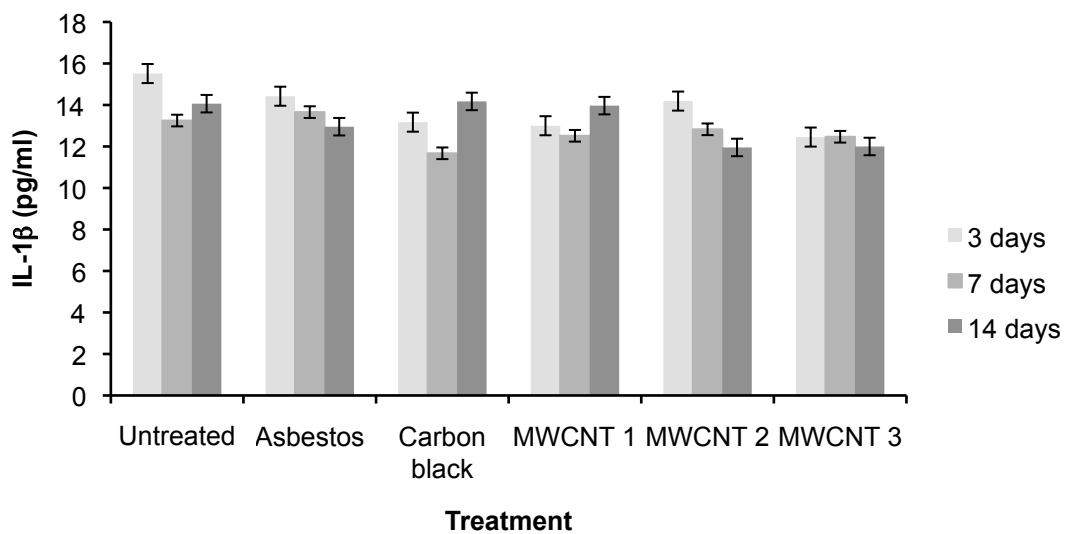

Supplement: Additional file 3 — IL-1β secretion after exposure to particulates in 3D cultures of macrophages. After 3, 7 and 14 days of exposure to A) 0.05 μg/ml (0.038 μg/cm2), B) 0.5 μg/ml (0.38 μg/cm2), or C) 2.5 μg/ml (1.9 μg/cm2) of particulates, secretion of IL-1 was evaluated in cell supernatants by ELISA. Bars represent the mean ± SD of triplicates. [file 1743-8977-8-17-S3.PDF]
