# Supplementary material for: A 3-dimensional in vitro model of epithelioid granulomas induced by high aspect ratio nanomaterials
Source: Part Fibre Toxicol. 2011 May 18;8:17. doi: 10.1186/1743-8977-8-17 (PMC3120675; doi:10.1186/1743-8977-8-17)
Supplement: Additional file 2 — M1 and M2 macrophage polarization in 2D and 3D cultures. This file contains a detailed description of the pro- and anti-inflammatory mediators induced in 2D and 3D cultures in response to LPS, IL-4 and IL-13 under serum-free conditions. [file 1743-8977-8-17-S2.DOC]

**Additional file 2**

**M1 and M2 macrophage polarization in 2D and 3D cultures.** Bone marrow-derived macrophages were treated with 100 ng/ml of lipopolysaccharide (LPS) and the M1 phenotype was assessed by TNF-α release, induction of iNOS expression, decreased MR expression and secretion as well as morphological changes [1, 2]. Although basal secretion levels of TNF-α are higher in 3D than in 2D cultures, at all time points evaluated (Figure S1A), this phenomenon is reversed upon treatment with LPS (Figure S1B) confirming that cellular attachment primes macrophages to LPS-induced TNF-α secretion [3]. The kinetics of LPS-induced TNF-α release is similar in 2D and 3D cultures with the highest level after 3 days, followed by a decline after 5 days (Figure S1A and B). Macrophage survival as assessed by DNA content (Figure S1C) and nuclear morphology (Figure S1G) does not decline until 10 days suggesting that the decrease in TNF-α secretion seen after day 3 is not due to LPS-induced cell death [4]. Following LPS exposure, transient iNOS expression [5] and decreased MR expression and release [6] occur only in 3D cultures (Figure S1E). Arg-1 induction is slightly evident in 3D cultures but not in 2D as analyzed using Western blot (Figure S1E). Expression of M1 and M2 markers during persistent LPS exposure was evaluated at the cellular level using indirect immunofluorescence and confocal microscopy (Figure S1F). Untreated cells in 2D cultures express basal levels of Arg-1 and iNOS after 3 days with co-localization in a subset of cells. In contrast, untreated in 3D cultures express a basal level of iNOS that decreases over time with no expression of Arg-1. Treatment with LPS induces persistent expression of Arg-1 and iNOS in 3D cultures with no evidence of cellular co-localization. iNOS and Arg-1 are only transiently induced in 2D cultureswith some cellular co-localization. LPS exposure induced similar patterns of MR and TNF-α expression in 2D and 3D cultures (Figure S1F). Low levels of expression of both markers are present in untreated cells, while LPS exposure decreases MR and increases TNF-α expression in both 2D and 3D cultures. The morphological characteristics of macrophages exposed to LPS, increased cellular size and spreading [2], are evident both in 2D and 3D cultures and are maintained up to 14 days (Figure S1F).

The anti-inflammatory phenotype was induced by exposure of BMDM to IL-4 or IL-13 for 14 days at 20 ng/ml. The M2 phenotype was assessed by induction of Arg-1 expression together with increased synthesis and release of MR and Ym1 [1, 7], indicating that these conditions induce M2 polarization in both 2D and 3D cultures. Although the expression profiles of M2 markers induced by IL-4 or IL-13 are similar, cells in 3D cultures show a more robust response (Figure S2A). After 14 days of continuous exposure to IL-4, 3D cultures show a higher number of cells co-localizing iNOS and Arg-1 than 2D cultures (Figure S2B). By indirect inmmunofluorescence and confocal microscopy, TNF-α levels are lower than in untreated cells (Figure S2B) and undetectable by ELISA assay (data not shown). Exposure to IL-4 or IL-13 also induces macrophages to fuse and form multinucleated giant cells [7]. Multinucleated giant cells are induced in both 2D and 3D cultures, although at a higher frequency in 2D than 3D cultures (70% and 20%, respectively). Both types of multinucleated giant cells, Langhans (Figure S2C- middle panel) and foreign body giant cells (Figure S2C-botton panel) are induced in 2D and 3D cultures. This is the first report of bone marrow-derived macrophages fusing in the absence of serum and attachment to a substrate [8].

In summary, 2D and 3D cultures of bone marrow-derived macrophages in serum-free medium are reproducible model systems to study macrophage activation since they recreate either a pro- or anti-inflammatory phenotype with expression of prototypical markers of M1/M2 polarization [1]. In 3D culture, sustained exposure to pro- or anti-inflammatory stimuli does not produce a shift in phenotype or deactivation [9] over the time course evaluated.

**Materials and Methods**

**Macrophage polarization**. To induce classical activation (M1), macrophages were treated with lipopolysaccharide (LPS) from *E.coli* 0111:B4 (Sigma) at a final concentration of 100 ng/ml in SFM, unless otherwise specified. To induce alternative activation (M2), IL-4 or IL-13 (R&D Systems, Minneapolis, MA) was added at a final concentration of 20 ng/ml.

**ELISA**. IL-1β and TNF-α levels in cell culture media were measured by ELISA (R&D Systems) following manufacturer’s protocol.

**Western blot analysis**. Cells were collected, washed, and resuspended in lysis buffer (2 % Triton X-100, 10 mM Tris-HCl, pH 8, 150 mM NaCl, 10 mM NaN3, 10 mM EDTA, 5 mM iodoacetamide, 2 mM phenylmethylsulfonyl fluoride, 1 g/ml pepstatin, and 1 M leupeptin). After incubation for 30 min on ice and vortexing every 10 min, cell lysates were centrifuged at 10,000 xg for 10 min at 4 °C in a Sorvall Legend Mach 1.6 R centrifuge (Kendro Laboratory Products Inc, Asheville, NC) and supernatants were transferred to a clean tube and stored at -80 °C until use.

Protein concentrations in cell lysates were determined using the DC protein assay (Bio-Rad). Samples (50 µg of total protein) were brought to equal volume with 5X Laemmli buffer to a final concentration of 2X, electrophoresed at 100 volts at 4 °C in 4-12 % SDS-polyacrylamide gradient gels (Bio-Rad), and transferred to Immobilon-P polyvinylidene difluoride membranes (PVDF, Millipore, Billerica, MA). Cell supernatants were concentrated 10 times in a Speed Vac Concentrator System (Savant Instrument. Inc., Farmingdale, NY), and stored at -20 °C. Nonreducing conditions were used for detection of MR. Membranes were blocked with 5 % non-fat milk/TBS for 30 min at room temperature, incubated overnight at 4 °C with primary antibodies against CD206/MR (AbD Serotec), iNOS, arginase-1 (BD Biosciences), Ym1 (StemCell Technologies, Vancouver, BC, Canada) or beta-actin (Cell Signaling Technology, Beverly, MA), washed 3 times with 0.1 % Triton X-100 in Tris- buffered saline solution followed by incubation for 2 hrs at room temperature with HRP-conjugated secondary antibody (Cell Signaling). After washing, specific bands were visualized using chemiluminescence (SuperSignal West Pico, Pierce/Thermo Scientific, Rockford, IL).

References

1. Mantovani A, Sica A, Sozzani S, Allavena P, Vecchi A, Locati M: **The chemokine system in diverse forms of macrophage activation and polarization.** *Trends Immunol* 2004, **25:**677-686.

2. Williams LM, Ridley AJ: **Lipopolysaccharide induces actin reorganization and tyrosine phosphorylation of Pyk2 and paxillin in monocytes and macrophages.** *J Immunol* 2000, **164:**2028-2036.

3. Haskill S, Johnson C, Eierman D, Becker S, Warren K: **Adherence induces selective mRNA expression of monocyte mediators and proto-oncogenes.** *J Immunol* 1988, **140:**1690-1694.

4. Xaus J, Comalada M, Valledor AF, Lloberas J, Lopez-Soriano F, Argiles JM, Bogdan C, Celada A: **LPS induces apoptosis in macrophages mostly through the autocrine production of TNF-alpha.** *Blood* 2000, **95:**3823-3831.

5. Vodovotz Y, Kwon NS, Pospischil M, Manning J, Paik J, Nathan C: **Inactivation of nitric oxide synthase after prolonged incubation of mouse macrophages with IFN-gamma and bacterial lipopolysaccharide.** *J Immunol* 1994, **152:**4110-4118.

6. Barish GD, Downes M, Alaynick WA, Yu RT, Ocampo CB, Bookout AL, Mangelsdorf DJ, Evans RM: **A Nuclear Receptor Atlas: macrophage activation.** *Mol Endocrinol* 2005, **19:**2466-2477.

7. Martinez FO, Helming L, Gordon S: **Alternative activation of macrophages: an immunologic functional perspective.** *Annu Rev Immunol* 2009, **27:**451-483.

8. Helming L, Gordon S: **Macrophage fusion induced by IL-4 alternative activation is a multistage process involving multiple target molecules.** *Eur J Immunol* 2007, **37:**33-42.

9. Mokart D, Kipnis E, Guerre-Berthelot P, Vey N, Capo C, Sannini A, Brun JP, Blache JL, Mege JL, Blaise D, Guery BP: **Monocyte deactivation in neutropenic acute respiratory distress syndrome patients treated with granulocyte colony-stimulating factor.** *Crit Care* 2008, **12:**R17.

**
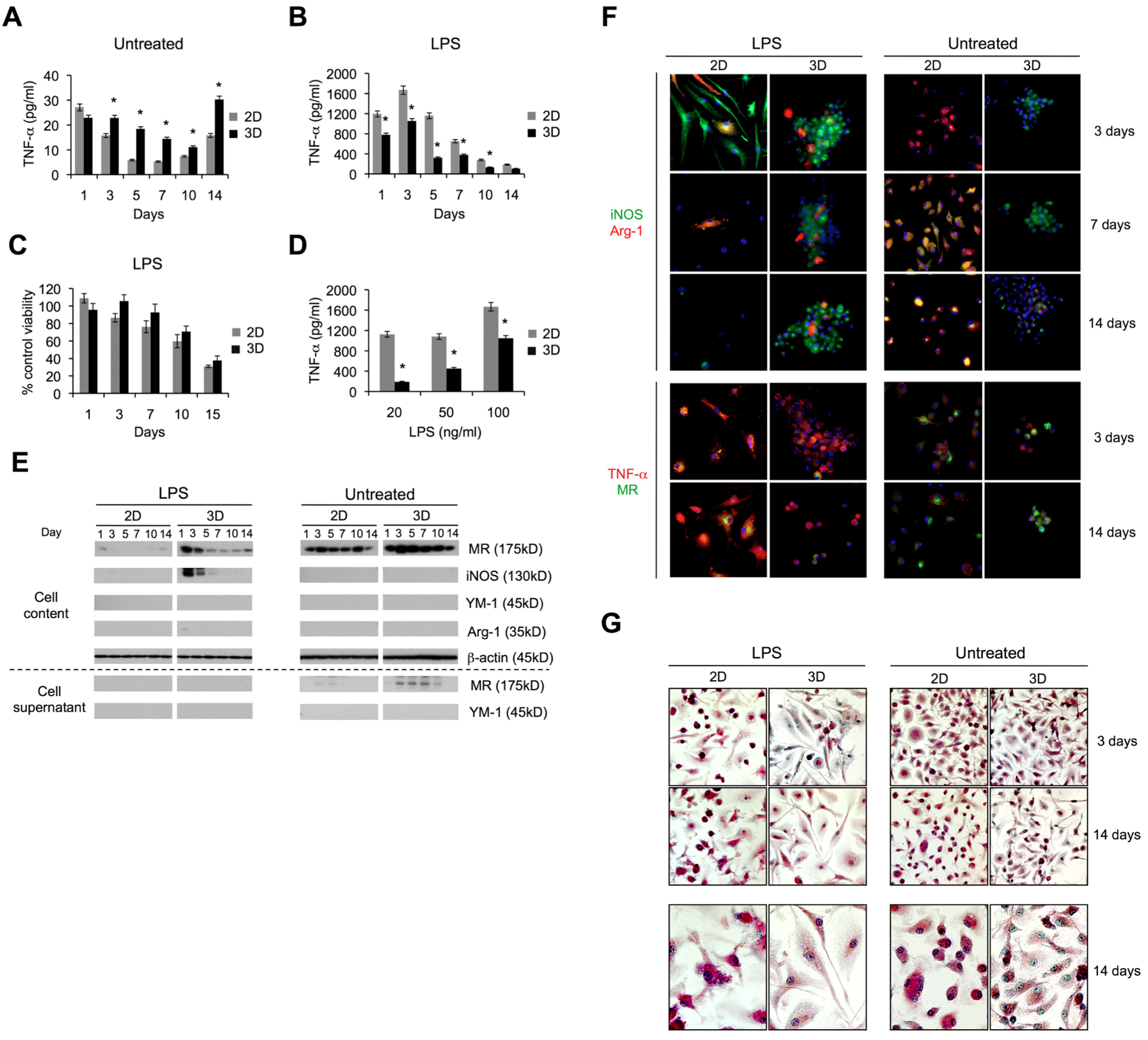
**

**Figure S1. LPS-induced TNF-α secretion, iNOS expression and MR down-regulation as markers of the proinflammatory phenotype and M1 polarization. A)** Basal levels of TNF-α secretion. **B)** Long-term exposure to 100 ng/ml of LPS induces time-dependent secretion of TNF-α. **C)** Survival of macrophages following exposure to 100 ng/ml LPS in 2D or 3D cultures. **D)** LPS-induced TNF-α release is dose-dependent and macrophages in 3D cultures are more responsive than in 2D cultures. Bars represent the mean ± SD of three separate determinations, **p*< 0.05, 2D vs 3D. **E)** Decreased MR expression and induction of iNOS at the protein level are characteristics of M1 polarization after exposure to 100 ng/ml LPS. **F)** Exposure to 100 ng/ml of LPS induces expression of TNF-α and iNOS, markers of M1 polarization both in 2D and 3D cultures (confocal microscopy showing patterns of colocalization with DAPI nuclear counterstain). Magnification: 400X. **G)** LPS (100 ng/ml) induces cell spreading and increased cell size in both 2D and 3D cultures. May-Grünwald-Giemsa stain. Magnification: 400X.

**
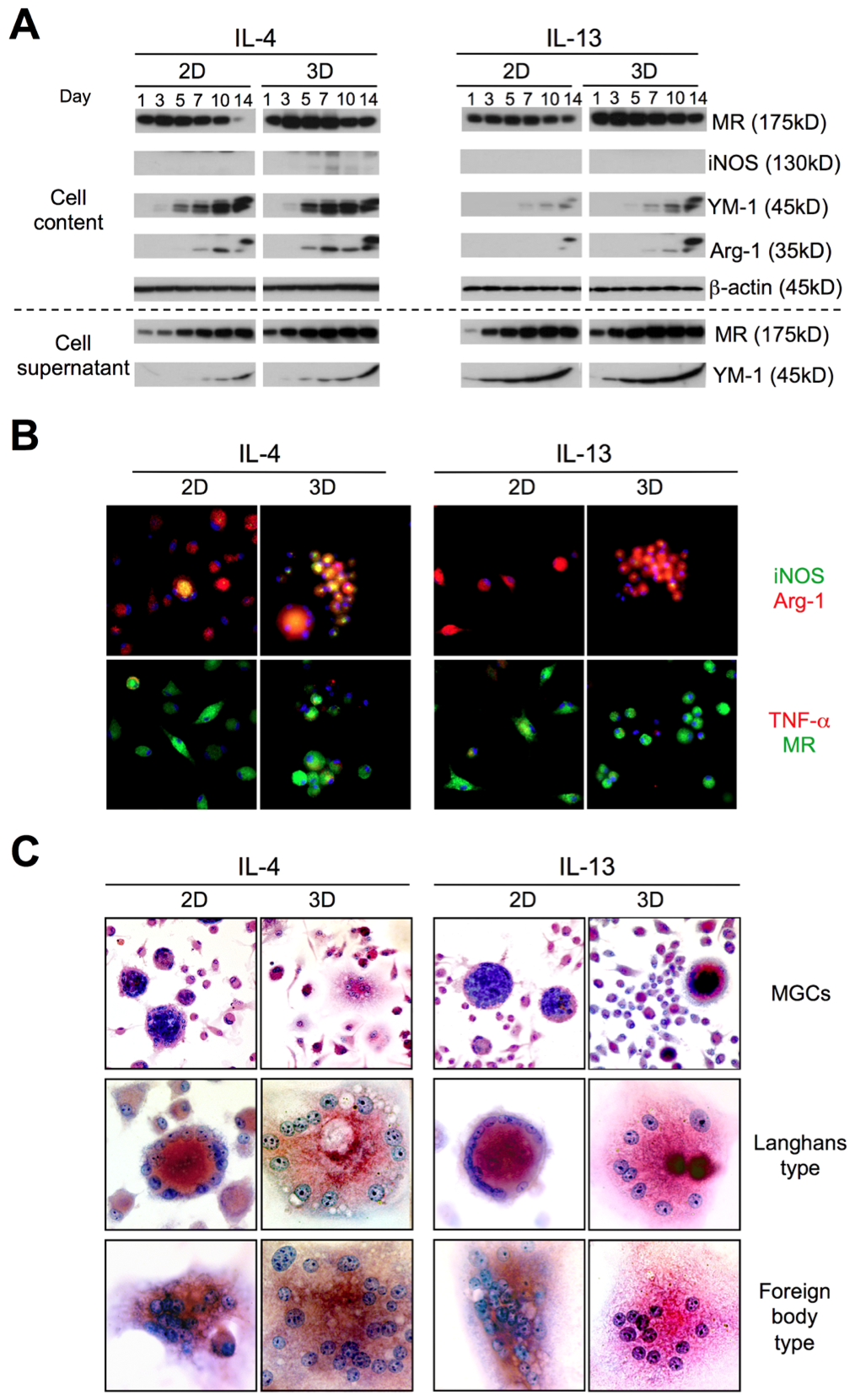
**

**Figure S2. IL- 4 and IL-13 induce an anti-inflammatory, profibrotic M2 phenotype and formation of multinucleated giant cells.** Macrophages were exposed to 20 ng/ml of IL-4 and IL-13. **A)** Time dependent up-regulation of Arg-1 expression as well as MR and Ym1 expression and secretion are indicative of M2 phenotype. **B)** IL-4 and IL-13 increase expression of M2 markers Arg-1 and MR after 14 days (confocal microscopy). Magnification: 400X. **C)** IL-4 and IL-13 induce formation of both types of MGC: Langhans (middle row) and foreign body (lower row) multinucleated giant cells. May-Grünwald-Giemsa stain. Magnification: 400X (top row) and 1000X (middle and bottom row).
