# Supplementary material for: A 3-dimensional in vitro model of epithelioid granulomas induced by high aspect ratio nanomaterials
Source: Part Fibre Toxicol. 2011 May 18;8:17. doi: 10.1186/1743-8977-8-17 (PMC3120675; doi:10.1186/1743-8977-8-17)
Supplement: Additional file 4 — Optimization of cell culture conditions for long-term survival of macrophages and characterization of cellular phenotype. This file includes the list of the cell culture media evaluated for optimal macrophage survival, comparison of cell survival between serum-free and serum-containing medium, and a phenotypic description of the cell population. [file 1743-8977-8-17-S4.DOC]

**Additional file 4**

**Optimization of cell culture conditions for long-term survival of macrophages and characterization of cellular phenotype.** DMEM supplemented with 10% FBS and 20% of conditioned medium (CM) from L-929 fibroblast-like cell line (M-CSF source), is conventionally used for both differentiation and maintenance of bone marrow-derived macrophages. Under this culture condition, upon differentiation, macrophage survival in 3D cultures rapidly declines and only 45% of the initial cell population is viable after 3 days, decreasing to 10 % after 16 days (Figure S1A). Several media formulations were tested to increase long-term survival of bone marrow-derived macrophages in 3D cultures (Table S1). X-Vivo-15 medium supplemented with 1X HL-1 and 20 ng/ml of M-CSF improved survival of 3D cultures to 100 % after 3 days and 47 % of viability after 16 days (Figure S1A). Cells in 3D survive significantly longer than adherent cultures (2D): after 15 days in culture, 30 % of the cells remain viable in 2D culture compared to 53% viability in 3D culture (Figure S1B).

To confirm macrophage phenotype, purity, and state of differentiation under these conditions, expression of specific surface markers was determined using flow cytometry (Figure S1C). After 3 days, the cellular population in 2D or 3D cultures shows similar expression of phenotypic markers, with no evidence of contaminating B-lymphocytes, pluripotent progenitor cells, or dendritic cells. The expression levels of CD11b, CD68, CD169 and F4/80 indicate that cells in both models express a monocyte/macrophage phenotype [1] without indication of transdifferentiation towards monocyte-derived dendritic cells.

**Materials and Methods**

**Characterization by flow cytometry**. After 3 days in culture, cells were washed twice with 0.1 % bovine serum albumin (BSA; Sigma) in PBS, incubated for 30 min at 4°C in blocking solution containing 10 % normal goat serum and/or anti-CD16/CD32 (Mouse BD Fc BlockTM, BD Biosciences) to saturate Fc receptors, 0.1 % BSA and 0.01 % sodium azide in PBS. Cells were then incubated with primary antibody or isotype control in blocking solution for 30 min at 4 °C. After 3 washes with 0.1 % BSA/0.0 1% sodium azide /PBS, cells were incubated with secondary antibody conjugated with Alexa 488 (Molecular Probes) for 30 min at 4 °C, washed, and fixed with fixation buffer (BD Biosciences). Cells were kept in 0.1 % BSA and 0.01 % sodium azide/ PBS at 4 °C and analyzed by flow cytometry (BD FACSCalibur)) using CellQuest software.

| Primary antibody | Company (catalog number), and isotype |
| --- | --- |
| CD11b | BD Biosciences (557396), rat IgG2b, k |
| CD11c | BD Biosciences (557400), american hamster IgG1 |
| CD33 | R&D Systems (AF2220) goat IGg |
| CD45r/B220 | BD Biosciences (557669), rat IgG2a, k |
| CD68 | AbD Serotec (MCA1957), rat IgG2a |
| CD117 | BD Biosciences (553868), rat IgG2b, k |
| CD169 | AbD Serotec (MCA884), rat IgG2a |
| F4/80 | AbD Serotec (MCA497), rat IgG2a |

**Additional table 1: Optimization of cell culture media:**

A total of 168 combinations were evaluated. In bold: the components of the final medium used. DMEM: Dulbecco's Modified Eagle Medium; M-SFM: macrophage serum-free media; FBS: fetal bovine serum; CM: conditional media from L-929 fibroblast; M-CSF: macrophage colony stimulation factor.

| **Cell culture media** | **Supplement** | **M-CSF source** |
| --- | --- | --- |
| DMEM^1^ | 10% FBS^3^ | 10% L-929 CM |
| RPMI 1640^1^ | 20% FBS^3^ | 20% L-929 CM |
| M-SFM^1^ | **1X HL-1**^2^ | 10 ng/ml M-CSF^4^ |
| AIM-V^1^ | 2X-HL-1^2^ | **20 ng/ml M-CSF**^4^ |
| HL-1^2^ |  |  |
| X-VIVO-10^2^ |  |  |
| **X-VIVO-15**^2^ |  |  |

^1^Invitrogen, Carlsbad, CA.

^2^Lonza Rockland Inc., Rockland, ME.

^3^Atlanta Biologicals, Lawrenceville, GA.

^4^R&D Systems, Minneapolis, MA.

**
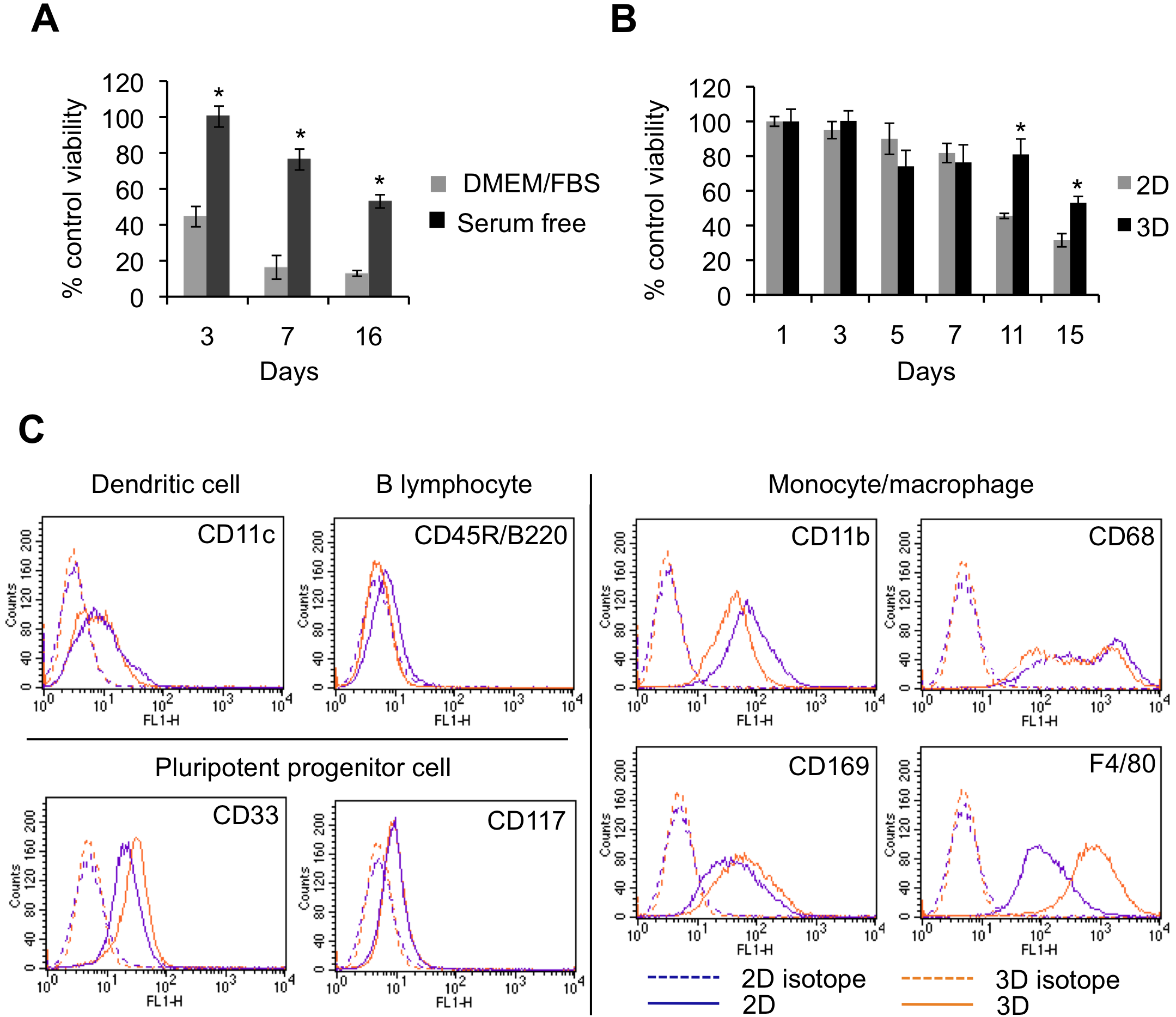
**

**Figure S1. Survival and phenotypic characteristics of macrophages** **in serum-free medium. A)** SFM improves the long-term survival of 3D cultures up to 50% when compared to DMEM/FBS. **B)** Under serum-free conditions, long-term viability is preserved in 3D compared to 2D cultures. Total DNA was quantified by PicoGreen assay after cellular digestion with Proteinase K as described in materials and methods. Bars represent the mean ± SD of three separate determinations; **p*< 0.05. **C)** Profile of phenotypic markers using flow cytometry analysis indicates absence of pluripotent progenitor cells, B lymphocytes, or DCs after 3 days in culture.
